# Impact of socioeconomic and environmental factors on atopic eczema and allergic rhinitis: a cross sectional study

**DOI:** 10.17179/excli2015-519

**Published:** 2015-09-11

**Authors:** Yasamin Torfi, Niloofar Bitarafan, Mehdi Rajabi

**Affiliations:** 1 Department of Clinical Pharmacy, Faculty of Pharmacy, Islamic Azad University of Pharmaceutical Sciences Branch, Tehran, Iran, Postal address: 19395-646

**Keywords:** allergic rhinitis, atopic eczema, prevalence, socioeconomic status

## Abstract

The prevalence of allergic rhinitis and atopic eczema is on the rise in recent decades. Many factors can be related to the development of these diseases. We aimed to investigate the association between socioeconomic status (SES), environmental risk factors and these conditions. In this study, the International Study of Asthma and Allergies in Childhood (ISAAC) questionnaire was translated and validated. Then it was used to determine the prevalence, severity and possible related factors for both diseases in 1904 schoolchildren aged 6-7 and 13-14 years from various regions of Tehran**. **The prevalence of rhinitis and eczema in the past year was 33.2 % and 8.2 %, respectively. The prevalence of rhinoconjunctivitis in the past year was 30 %. The risk factors such as birth order, nursery attendance, pet ownership, past allergic experiences as well as some SES factors were associated with both conditions. The prevalence of allergic rhinitis and atopic eczema was on the rise in comparison to the previous studies and SES as well as environmental factors are thought to be associated with the prevalence of these conditions.

## Introduction

Asthma, rhinitis and eczema are common public health problems throughout the world, affecting the quality of life of the patients especially children. According to World Health Organization (WHO) statistics, 400 millions of subjects in the world suffer from rhinitis and 15-30 % of children from eczema (Ansotegui et al., 2011[2]). ISAAC findings have shown the prevalence of these conditions is rising dramatically in both developed and developing countries (ISAAC, 2012[12]). Genetic and environmental factors are known to play a role in the development of allergic diseases. There are several environmental risk factors for allergic diseases including tobacco smoke, poor housing condition and air pollution. At least one of these factors can be associated with socioeconomic status (SES). There are some reports suggesting that allergic diseases may be more prevalent in lower SES while some reports have shown that low SES can be a protective factor for atopic diseases as suggested by hygiene hypothesis (Ansotegui et al., 2011[2]; Uphoff et al., 2014[18]). Therefore the association between SES and allergic diseases remains controversial. 

There has been no study conducted on the relation between SES and the development of allergic diseases in Tehran (as part of ISAAC investigation), therefore, this study was designed to assess the prevalence of self-reported allergic rhinitis and atopic eczema among primary and secondary school students and its relation to SES and other risk factors.

## Materials and Methods

This cross-sectional study was carried out from January 2013 to January 2014. The study population consisted of schoolchildren aged 6-7 and 13-14 years in the city of Tehran. The standardized questionnaire of ISAAC Protocol was translated into Persian language and validated by several pilot studies. The final questionnaire was randomly distributed amongst primary and secondary public schools located in different parts of the city. The parents were informed and instructed how to fill the questionnaire at home and returned in the predefined time. 

The ISAAC standard questionnaire consisted of two sections, involving questions relating to the prevalence and severity of diseases and risk factors. Positive answers to the question ”Has your child had itchy rash affected the folds of the elbows, behind the knees, in front of the ankles, around the neck, ears or eyes in the last 12 months?” were considered to be atopic eczema. Positive answers to the question “In the last 12 months, has your child had a problem with sneezing or a running or blocked nose when she/he did not have a cold or flu?” were considered to be as allergic rhinitis. The severity of these conditions was assessed by interference with patients' daily activities for rhinitis and patients' sleep disturbance for eczema. 

From approximately 4000 distributed questionnaires, 2000 were collected. Missing and inconsistent responses were excluded from analysis. Finally, 1904 questionnaires were analyzed using the statistical software SPSS, Version 16. The prevalence of the symptoms was calculated and comparison between groups was made using the chi-squared test and logistic regression. P-value < 0.05 was considered significant. A number of variables including; gender, age, birth order, number of children, infectious diseases (measles, pertussis, tuberculosis and worm infection), acetaminophen and antibiotic consumption, breastfeeding, nursery attendance, pet ownership, parental history of allergic disease (asthma, rhinitis and eczema) and parental smoking habit were investigated. Comorbidities between allergic diseases (asthma, rhinitis and eczema) were also assessed. SES of participants was defined as parental education level, parental job type and family monthly income. The study was approved by ethics committee of Islamic Azad University of Pharmaceutical Sciences Branch (Reference number: 5248).

## Results

Among the 1904 participants, 44.7 % were boys and 55.3 % were girls. 59.3 % were 6-7 year old and 40.7 % were 13-14 year old. 36.1 % of participants were in northern, 28.8 % in southern, 19.7 % in western, 5.9 % in eastern and 9.4 % in the central part of the city. The prevalence of self-reported allergic rhinitis and atopic eczema in the past 12 months was 33.2 % and 8.2 %, respectively. Table 1(Tab. 1) shows the comparison between the prevalence rate of rhinitis and eczema in various cities of Iran and the current study based on ISAAC protocol. Among students with eczema, 61.6 % of sufferers reported that their rash had cleared completely during the last 12 months. Eczema symptoms caused school absence in 5.2 % and sleep disturbance in 24.1 % of the patients. 55.3 % patients had visited specialist and 64.7 % had used medication for the treatment of eczema symptoms. In patients with rhinitis, 30 % had conjunctivitis concurrently. Rhinitis symptoms caused school absence in 26.1 % and frequent interference with daily activities in 2.7 % of the patients. 58.1 % patients had visited specialist and 61.6 % had used medication for the treatment of the rhinitis symptoms. The comparison between the severity of rhinitis and eczema symptoms in the current study and the previous studies in Iran is presented in Table 2(Tab. 2). 

There was no association between gender, age, number of children, infectious diseases, acetaminophen use (during past year and first year of life), antibiotic use (during first year of life), pet exposure (during first year of life) and breastfeeding with rhinitis and eczema. However, the prevalence of rhinitis was significantly associated with parental smoking at present (OR (95 % CI) 1.36 (1.05-1.77). Those students who attended nursery at the earlier age had a higher chance of developing rhinitis as compared with the ones who did not attend the nursery 1.33 (1.10-1.62). Rhinitis was more prevalent among the first born children 1.42 (1.16-1.73), but there was no association between these factors and eczema. Pet exposure at present time was also associated with symptoms of eczema 1.92 (1.30-2.82), whereas no association was found between this factor and rhinitis. Parental history of allergic diseases (asthma, rhinitis and eczema) had a substantial association with both allergic rhinitis and atopic eczema. Table 3(Tab. 3) shows the relationship between symptoms of rhinitis, eczema and the risk factors. 

Among SES factors, rhinitis was appreciably associated with father and mother's educational level. With reduction in father's educational level, the risk of development of rhinitis increased and also there was a higher prevalence of rhinitis among patients with illiterate mothers. A significant positive relationship between the prevalence of rhinitis and eczema and family monthly income was found. Table 4(Tab. 4) shows the association between SES and these conditions. 

**Figure 1:**
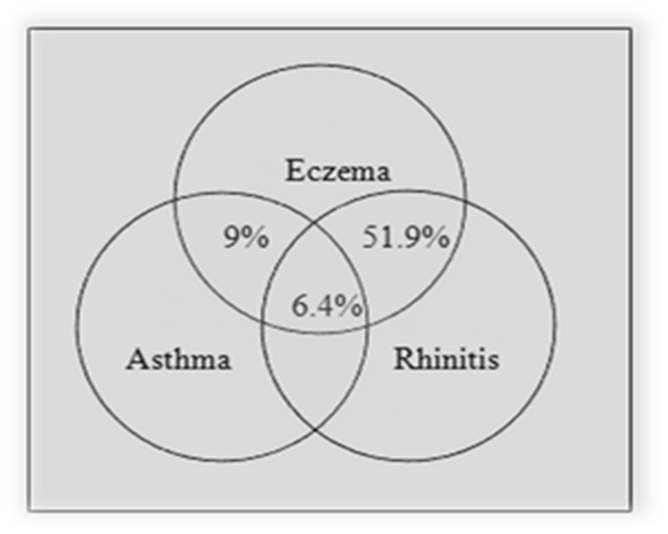
The association between eczema and allergic diseases

**Figure 2:**
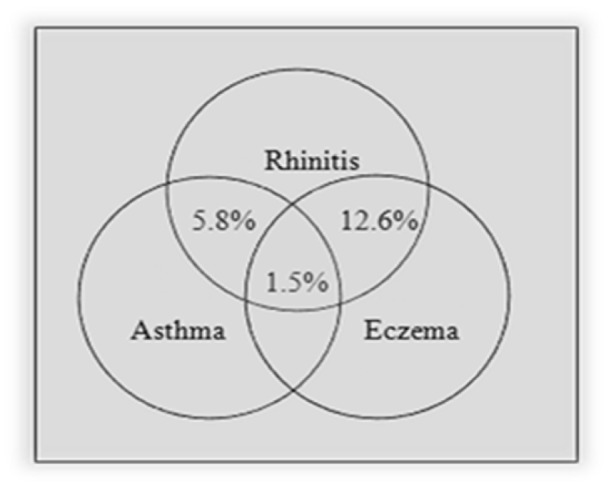
The association between rhinitis and allergic diseases

There was a significant association between rhinitis and other allergic diseases (P-value < 0.005, 4.09 (2.33-7.17) for asthma and P-value < 0.005, 2.35 (1.67-3.27) for eczema). The same association was also observed in eczema (P-value < 0.005, 4.14 (2.20-7.77) for asthma and P-value < 0.005, 2.35 (1.68-3.27) for rhinitis). The comorbidities of asthma, rhinitis and eczema are presented in Figures 1(Fig. 1) and 2(Fig. 2). As presented in these figures, amongst patients with rhinitis, 5.8 % had asthma, 12.6 % had eczema and 1.5 % had asthma and eczema concurrently. 9 % of patients with eczema had asthma, 51.9 % had rhinitis and 6.4 % had asthma and rhinitis concurrently. 

## Discussion

There have been numerous studies investigating the prevalence of allergic rhinitis and atopic eczema in various parts of the world, using different methods. The international study of asthma and allergic diseases in childhood program has assessed the prevalence of allergic rhinitis and atopic eczema in children by standard methods. According to ISAAC phase one findings, the worldwide variations in prevalence rate of allergic diseases may be related to environmental factors (Beasley et al., 1998[6]). ISAAC phase three findings have reported an increase in all allergic disorders in most centers (Asher et al., 2006[3]). In this study we evaluated the allergic rhinitis and atopic eczema prevalence and its association with environmental factors and SES, using ISAAC questionnaire. The prevalence rates of rhinitis, rhinoconjunctivitis and eczema in the past 12 months were 33.2 %, 30 % and 8.2 %, respectively. Our findings show that the prevalence and severity of rhinitis, rhinoconjunctivitis and eczema has increased in comparison to previous studies based on ISAAC protocol in Tehran (ISAAC, 2012[12]).

A few epidemiological studies about the relationship between environmental risk factors and allergic diseases have been conducted in Iran. In this study no differences were found in the prevalence of rhinitis and eczema between males and females as the findings of J. Batlls-Garridoa et al. (2010[5]), while an association between gender and these conditions were found in another study (Chereches-Panta et al., 2011[9]).

According to hygiene hypothesis, nursery attendance, crowded families and having older siblings are protective factors against atopic diseases due to higher exposure to infections in early life. The protective effect of these three factors has been observed in some studies (De Meer et al., 2005[10]; Strachan et al., 2015[16]; Zekveld et al., 2006[21]). In line with this hypothesis, first born children tend to have a higher chance of developing rhinitis as we observed in our study. Despite this hypothesis, we found a positive association between nursery attendance and rhinitis which is keeping with Pekkanen et al. (1999[14]) findings. In the present study no significant relationship was found between family size and atopic diseases.

WHO estimates that nearly 700 million, or almost half of the world's children, breathe air which maybe polluted by tobacco smoke, particularly at home. The link between second-hand smoke and several health outcomes, such as asthma, have long been established (WHO, 2015[19]). In this study, we found that parental smoking at “the present” was a risk factor for rhinitis in children. A review of 63 studies about the association between parental smoking and the prevalence of allergic rhinitis in children, have confirmed this association (Saulyte et al., 2014[15]). 

Several studies have been conducted to evaluate the early life exposure to acetaminophen and/or antibiotics and its association with the development of allergic diseases. From the Phase 3 of ISAAC findings, acetaminophen and antibiotic use represented important risk factors for the development of allergic diseases (Foliaki et al., 2009[11]; Beasley et al., 2011[6]). In contrast to these findings, there was not a significant correlation between acetaminophen and antibiotics use and the prevalence of rhinitis and eczema in our study. 

Studies investigating the association between breastfeeding and allergic diseases have produced conflicting results. Some studies have shown a protective effect (Kull et al., 2010[13]) while others including ISAAC findings have found no relationship (Björkstén et al., 2011[8]). Similar to ISAAC results, there was no association between breastfeeding and rhinitis and eczema in our study. 

As for the comorbidity between atopic diseases (asthma, rhinitis and eczema), many reports have demonstrated all three disorders had significant correlations with each other (Yuksel et al., 2008[20]; Ballardini et al., 2012[4]). In this study we found the co-existence of rhinitis, asthma and eczema. Numerous studies have shown that allergic diseases have a strong genetic component. This risk seems to be present regardless to the type of allergic diseases of family members (Tanaka et al., 2007[17]). 

A number of previous studies have shown a significant correlation between exposure to pets and eczema, mostly indicating a positive relationship (Ahlbom et al., 1998[1]). Similarly, in our study such relationship was observed. 

The evidence on the relationship between SES and asthma and allergies has been conflicting. The evidence from a systematic review of 183 articles suggests that rhinitis and eczema are more prevalent in higher SES groups (Uphoff et al., 2014[18]). In the present study, rhinitis and eczema were more prevalent in higher income families. This finding can be explained by the hygiene hypothesis. The prevalence of rhinitis had an inverse association with paternal educational level. One possible explanation for this association is that, the environmental factors such as tobacco smoke, air pollution, poor housing conditions and diet can be associated with lower educational level. 

This study has several strengths. First, the ISAAC questionnaire used in this study has been validated worldwide so our data can be easily compared with other studies based on ISAAC protocol. Another advantage of the study is the large sample size from several regions of the city. On the other hand, the limitations of the study could be that the prevalence of the diseases was assessed by self-reported questionnaire only. Clinical assessment by an experienced specialist is regarded as the best way to diagnose these conditions and this is not practical in cross-sectional studies with the large sample sizes. Moreover, the questionnaire data was completed by the parents of the children and this could lead to recall bias for some of the variables. In conclusion, in our study population, we demonstrated an increase in prevalence and severity of allergic rhinitis and atopic eczema in comparison to previous studies. Personal and parental history of allergic disease was recognized as risk factors for rhinitis and eczema. Significant risk factors were first born child, nursery attendance and parental smoking for rhinitis and pet ownership for eczema. The relationship between rhinitis and eczema and SES needs to be further explored with more focus on environmental factors in the long-term follow up or case-control studies.

## Acknowledgements

The author and the co-authors would like to express their appreciation to all students, parents and those schools for participating in our investigation. This study was one of the largest investigations in its own right in Iran and without their help it could not be possible.

## Conflict of interest

The authors declare that they have no conflict of interest. Indeed all authors would declare there is no conflict of interest with any manufacturer, drugs or any institutes as far as this manuscript is concerned.

## Figures and Tables

**Table 1:**
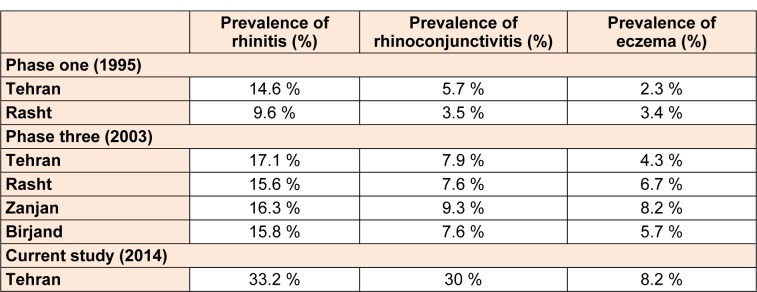
Comparison between prevalence of rhinitis and eczema in past 12 months between various cities of Iran based on ISAAC protocol

**Table 2:**
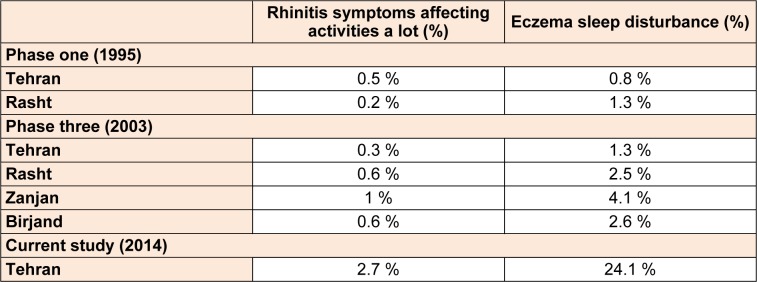
Comparison between severity of rhinitis and eczema in past 12 months between various cities of Iran based on ISAAC protocol

**Table 3:**
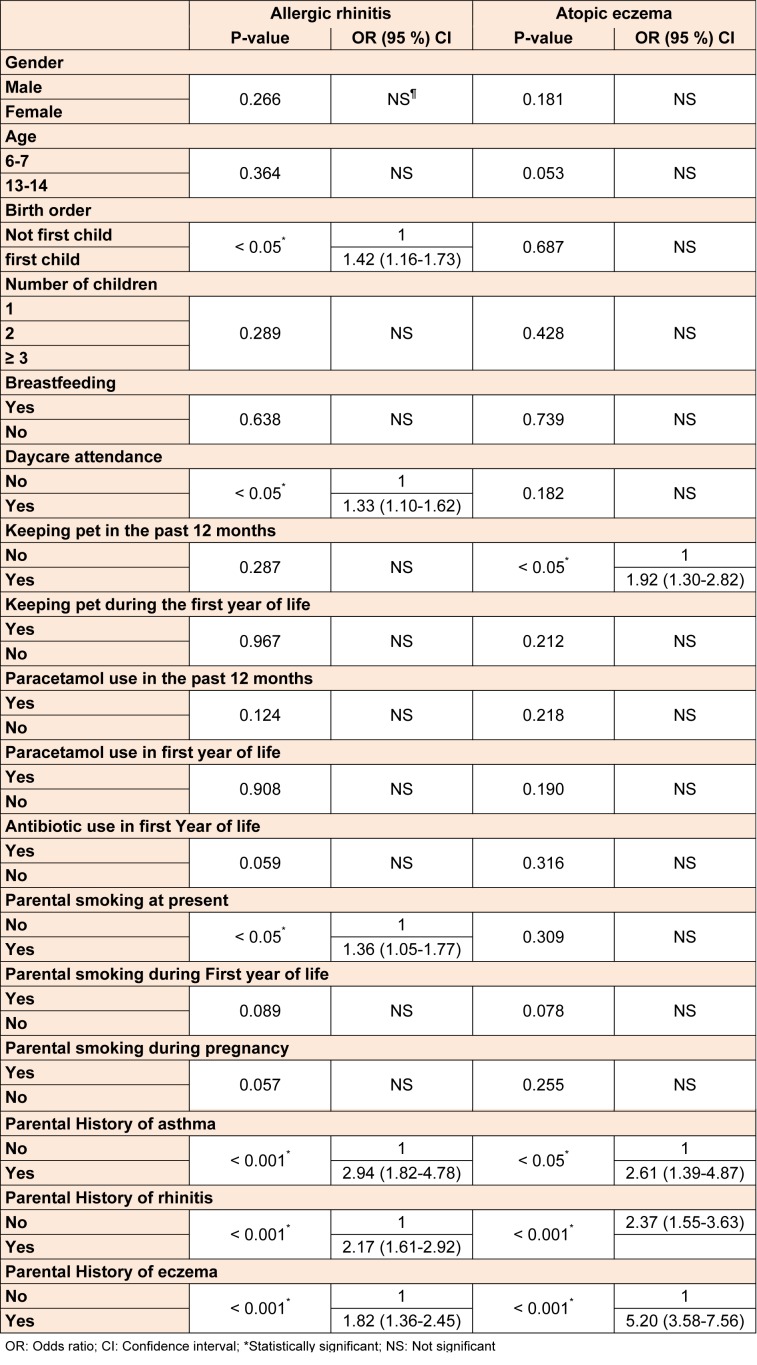
The association between risk factors and rhinitis and eczema

**Table 4:**
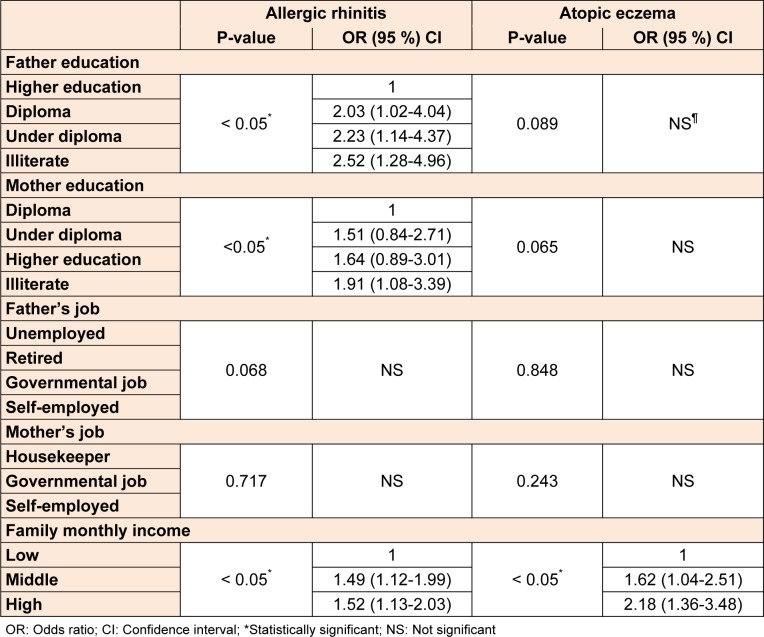
The association between socioeconomic status and rhinitis and eczema
